# Enhanced Energy Storage Properties of La-Doped Sr_0.6_Ba_0.4_Nb_2_O_6_ Relaxor Ferroelectric Ceramics Prepared by Spark Plasma Sintering

**DOI:** 10.3390/ma15124360

**Published:** 2022-06-20

**Authors:** Yingying Zhao, Xiao Liu, Xiaoyu Zhang, Huiling Du

**Affiliations:** 1 College of Materials Science and Engineering, Xi’an University of Science and Technology, Xi’an 710054, China; liuxiao@xust.edu.cn; 2 State Key Laboratory for Mechanical Behavior of Materials, School of Materials Science and Engineering, Xi’an Jiaotong University, Xi’an 710049, China; xyzhang918@163.com

**Keywords:** spark plasma sintering, strontium barium niobate, energy storage performance, relaxor ferroelectric ceramics

## Abstract

In this work, La-doped Sr_0.6_Ba_0.4_Nb_2_O_6_ ferroelectric ceramics were fabricated by the conventional solid state reaction method (CS) and spark plasma sintering (SPS), respectively. The microstructure, phase structure, dielectric properties, relaxor behavior, ferroelectric and energy storage properties were investigated and compared to indicate the effects of spark plasma sintering on their performances. The results show that the grain shape changes from columnar to isometric crystal and the grain size decreases obviously after spark plasma sintering. The dielectric constant of the CS sample and the SPS sample both show a typical relaxor behavior with obvious frequency dispersion. The diffusion parameters (γ) of both CS sample and SPS sample are close to 2 and all the samples present slim polarization–electric (P-E) loops, which verify the relaxor behavior. Moreover, the breakdown strength, *E*_b_, and discharge energy storage density, *W*_rec_, of La-doped Sr_0.6_Ba_0.4_Nb_2_O_6_ ferroelectric ceramics prepared by SPS are improved significantly. This work provides guidance for improving the energy storage performance of ferroelectric ceramics with tungsten bronze structures by decreasing the grain size through adopting a different sintering method.

## 1. Introduction

With the environmental problem caused by the consumption of fossil energy, renewable energy and energy storage technology need to be developed urgently. Due to the ultrafast charge and discharge rate, high power density and good cycling performance of dielectric capacitors, they have great potential in energy storage devices, including smart power grids, impulse power, electric vehicles and so on [1,2,3,4]. There are four main categories of dielectric energy materials, including linear dielectrics, ferroelectrics (FE), relaxor ferroelectrics (RFE) and antiferroelectrics (AFE). Among them, ferroelectric and antiferroelectric ceramics exhibit great potential in energy-storage fields due to small residue polarization *P*_r_ [5,6,7]. However, most antiferroelectrics with excellent properties contain lead, which would cause serious environment problems. Other AFE systems like AgNbO_3_-based materials show inferior anti-fatigue performance. Consequently, RFE ceramics have unique advantages when applied in energy storage devices because they have a slim P-E loop.

Strontium barium niobate, SBN (Sr_x_Ba_1−x_Nb_2_O_6_, 0.25 ≤ x ≤ 0.75), as a lead-free relaxor ferroelectric material, is a promising lead-free alternative. SBN has an unfilled tetragonal tungsten bronze structure with a formula of (A1)_2_(A2)_4_C_4_(B1)_2_(B2)_8_O_30_. The B1 and B2 sites are occupied by Nb ions, which form NbO_6_ octahedra, combining O ions [8,9]. Sr ions occupy both the A1 site and the larger A2 site, whereas the larger Ba ions occupy the A2 site only. The smaller C site remains empty [10,11]. Moreover, SBN ceramics transition from normal ferroelectrics to relaxor ferroelectrics when the Sr/Ba increases [10]. It is well known that *E*_b_ has a greater influence on the energy storage density compared to the permittivity [12], and the *E*_b_ is closely related to grain size. Consequently, there are many studies about refining the grain size of ferroelectrics by introducing different elements and changing the sintering method [13,14,15]. Song et al. introduced a glass addition and prepared the SBN ceramics by conventional solid state reaction method, which obtained a decreased grain size and improved breakdown strength [16]. Xiu et al. prepared SBN glass-ceramics and refined the grain size by introducing a glass addition [17]. Moreover, it has been reported that the grain size can also be refined by changing the sintering method. Zhao et al. prepared Bi_0.5_Na_0.5_TiO_3_ (BNT)-based ceramics and multilayer ceramic capacitors by a two-step sintering method and obtained obviously decreased grain size [18]. Huang et al. prepared Ba_0.4_Sr_0.6_TiO_3_@SiO_2_ dense ceramics via the spark plasma sintering method and obtained sub-micron grain size [19]. Spark plasma sintering (SPS) technology has been employed in many systems, including Ba_0.4_Sr_0.6_TiO_3_, Pb(Zr,Ti)O_3_ and so on. During SPS process, direct current, high mechanical pressure and low voltage are applied, resulting in the ultrahigh heating and cooling rates and a very short sintering time. Thus, the microstructures and grains growth of ceramics prepared by SPS can be controlled well [19,20,21,22].

While due to the grains of SBN ceramics often are anisometric and columnar, the microstructure of SBN ceramics prepared by CS is not very dense, as a result of which the breakdown strength and the energy storage performance are poor. Therefore, in this work, the La-doped Sr_0.6_Ba_0.4_Nb_2_O_6_ ferroelectric ceramics were prepared by CS and SPS, respectively. The doping of La^3+^ can reduce the ferroelectric domain size further and enhance the relaxor behavior. The morphology, dielectric response, relaxor behavior and energy storage properties are compared. With the SPS sintering method, the grain size decreases obviously, and the grain shape changes from a columnar to an isometric crystal. Consequently, increased breakdown strength and higher energy storage efficiency have been obtained.

## 2. Experimental Procedures

The conventional solid state reaction method (CS) and spark plasma sintering (SPS) technology were used to prepare ${\mathrm{Sr}}_{0.585}{\mathrm{Ba}}_{0.4}{\mathrm{La}}_{0.02}{\mathrm{Nb}}_{2}O_{6}\;$ (SBN60-2La) ceramics. The raw materials were BaCO_3_ (99.9%), SrCO_3_ (99.9%), La_2_O_3_ (99.9%) and Nb_2_O_5_ (99.9%), which were all bought from the Alfa Aesar company. The samples are denoted as CS sample and SPS sample, respectively. The CS process was reported in a previous article [10] and the SBN60-2La samples in this work were sintered at 1350 °C for 4 h. For the SPS sample, the SBN60-2La powders were presintered by CS and then put into a graphite die with a diameter of 10 mm. In order to avoid contact between the powder and the die, a graphite foil was used in the SPS sintering process. Then the powders were sintered by SPS at 1000 °C for 5 min. Finally, the samples sintered by SPS were annealed at 900 °C for 2 h to remove the residual carbon caused by the graphite foil and die in SPS sintering process.

The phase structure of all the samples were detected by X-ray diffraction (XRD, X’Pert diffractometer with Cu Kα λ−0.15406 nm) at room temperature. A scanning electron microscope (SEM, Hitachi S-2700) was used to detect the surface microstructure and fracture microstructure. In order to detect the surface microstructure, the SBN60-2La ceramics prepared by SPS were thermal etched at 1000 °C for 20 min and the SBN60-2La ceramics prepared by CS were thermal etched at 1250 °C for 10 min. The HIOKI LCR Hitester was used to measure the dependence of the dielectric response within the temperature range of −50 °C–200 °C. The Precision Premier II (from Radiant Company) with a high voltage amplifier was used to characterize the P-E loops.

## 3. Results and Discussions

**Figure 1:**
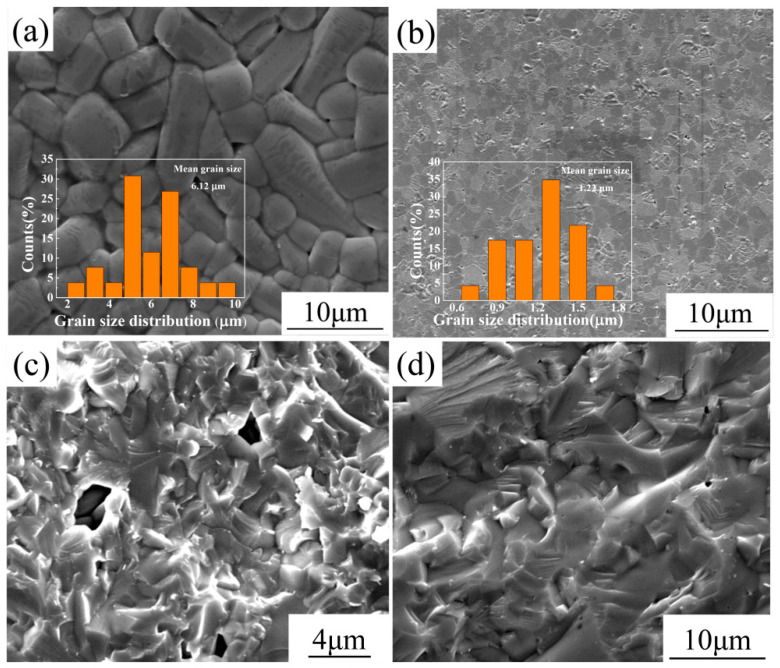
The surface morphologies and the fracture surface morphologies of SBN60-2La ceramics prepared by CS and SPS: (**a**) CS, (**b**) SPS, (**c**) CS, (**d**) SPS.

Figure 1 depicts the comparison between SEM images of SBN60-2La ceramics prepared by CS and SPS. The surface morphologies and the fracture surface morphologies of the CS sample and SPS sample are shown in Figure 1a–d, respectively. Obviously, it can be seen from Figure 1a,b that the CS samples show anisometric and columnar shaped grains, while the grains of SPS samples are isometric and the size of the SPS samples is much smaller than that of the CS samples. The average grain size of the SPS sample is 1.22 μm, while the CS sample has an average grain size of 6.12 μm. Moreover, in order to investigate the detailed microstructure, the fracture surface morphologies of the CS sample and SPS sample are shown in Figure 1c,d, respectively. It can be seen that the microstructure of SBN60-2La, prepared by SPS, is denser. Due to the columnar shape grain of CS sample, the microstructure is not dense enough and obvious pores between the grains can be seen in Figure 1c, while the SPS sample in Figure 1d shows a much more compact microstructure with a few very small pores. This is attributed to the fast sintering rate and short sintering time of the SPS sintering method.

**Figure 2:**
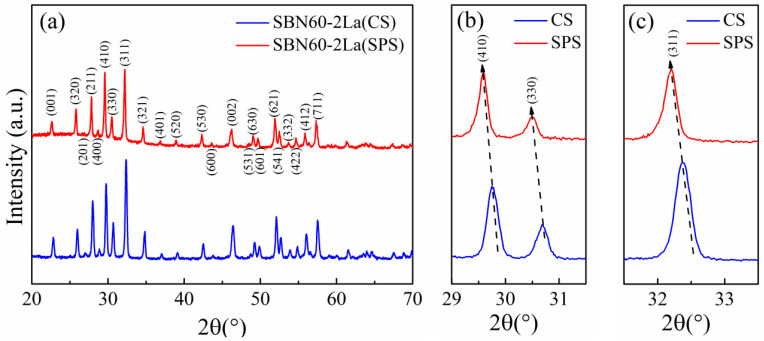
(**a**) X-ray diffraction patterns at room temperature, (**b**) (410) and (330) peak profiles, (**c**) (311) peak profiles for the CS sample and SPS sample.

The XRD patterns of the CS sample and SPS sample are illustrated in Figure 2. It can be seen from Figure 2a that both the CS sample and SPS sample show a pure tetragonal tungsten bronze structure without an impurity phase, which indicates that the SBN60-2La ceramics prepared by SPS has good crystallinity. The (410), (330) and (311) peak profiles are presented in Figure 2b,c. It can be seen that the peak of SPS sample shows slight shifts towards lower 2θ values, indicating a gradual increase in lattice parameters and unit cell volume. This maybe because the carbon element caused by the graphite foil and die used in SPS sintering may enter into the SBN60-2La sample.

**Figure 3:**
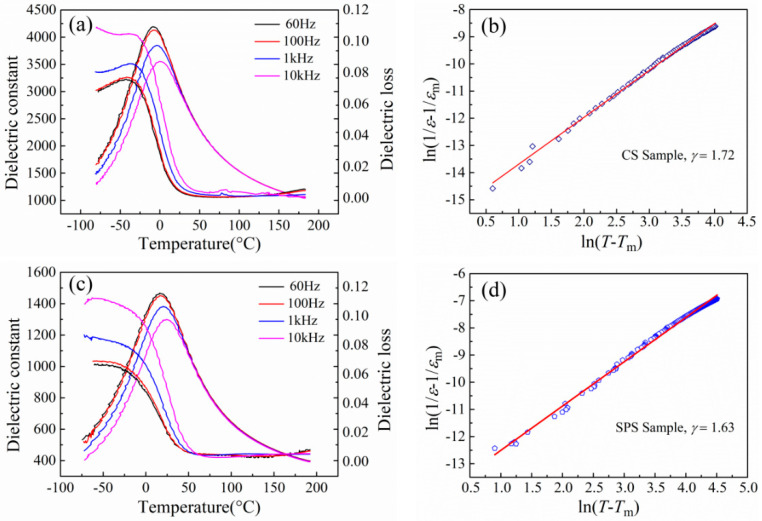
The dielectric properties of SBN60-2La ceramics: the temperature dependence of dielectric properties of (**a**) CS sample; (**b**) SPS sample, plots of ln(1/*ε* − 1/*ε*_m_) versus ln(*T* − *T*_m_) at 1 kHz; (**c**) CS sample; (**d**) SPS sample.

Figure 3 depicts the temperature dependence of dielectric properties and the relaxor properties of SBN60-2La ceramics prepared by CS and SPS. It can be seen from Figure 3a,c that when the temperature is below the temperature (*T*_m_) of the maximum dielectric constant, both the CS sample and SPS sample show an distinct frequency-dependent behavior. With the frequency increasing, the *T*_m_ shifts towards higher temperature. Both the CS sample and SPS sample show typical relaxor behavior with obvious frequency dispersion. Moreover, it can be seen that the dielectric constant of SPS samples is much smaller than that of CS samples. This phenomenon is closely related with the grain size. The grain size effects on the dielectric properties of perovskite ferroelectric ceramics have been widely reported. The ceramics with smaller grain size show a lower dielectric constant value and the samples with uniform grain size distribution show a higher dielectric constant value when the grain size is almost same [23,24,25]. So, it can be concluded that the smaller dielectric constant value of SPS samples is attributed to the smaller grain size of SPS sample. Moreover, the dielectric loss of both the CS sample and SPS sample shows a high value below the *T*_m_, and the dielectric loss decreases rapidly to a very small value when the temperature increases.

In order to investigate the diffuseness degree of the CS sample and SPS sample, the modified Curie–Weiss law as follows was used to fit:

(1/*ε* − 1/*ε*_m_) = (*T* − *T*_m_)*^γ^*/*C* (1)

where *ε*_m_ represents the maximum value of the dielectric permittivity at *T*_m_, the *γ* (1 ≤ *γ* ≤ 2) value indicates the diffuseness and *C* is the Curie constant. Normally, *γ* = 1 suggests normal ferroelectric with a Curie–Weiss behavior, while *γ* = 2 represents for an ideal relaxor ferroelectric. In order to investigate the influence of the SPS sintering method on the relaxor behavior of the SBN60-2La ceramics, the plots of ln(1/*ε* − 1/*ε*_m_) as a function of ln(*T* − *T*_m_) at 1 kHz of CS sample and SPS sample are shown in Figure 3b,d, respectively. The diffuseness value *γ* of CS sample and SPS sample are 1.63 and 1.72, respectively, which are both close to 2. This indicates that both the CS sample and SPS sample show a typical diffuse transition. The *γ* value of SPS sample is close to that of the CS sample, which indicates that the SPS sintering method has little influence on the relaxor behavior of the SBN60-2La ceramics.

The P-E loops of SBN60-2La ceramics prepared by CS and SPS are exhibited in Figure 3a,b, respectively. It can be observed that La-doped SBN ferroelectric ceramics show a very slim P-E loop, which verifies the relaxor feature. This is consistent with the results obtained in the dielectric measurement. Based on the P-E loops given in Figure 3a,b, the total energy storage density (*W*_total_), the recoverable energy storage density (*W*_rec_) and the energy storage efficiency (*η*) could be calculated by using the following formula (2 and 5):

(2) $$W_{\mathrm{total}}=\int_{0}^{P_{\max}}EdP$$

(3) $$W_{\mathrm{rec}}=\int_{P_{r}}^{P_{\max}}EdP$$

(4) $${\eta =W}_{\mathrm{rec}}{/W}_{\mathrm{total}}$$

**Figure 4:**
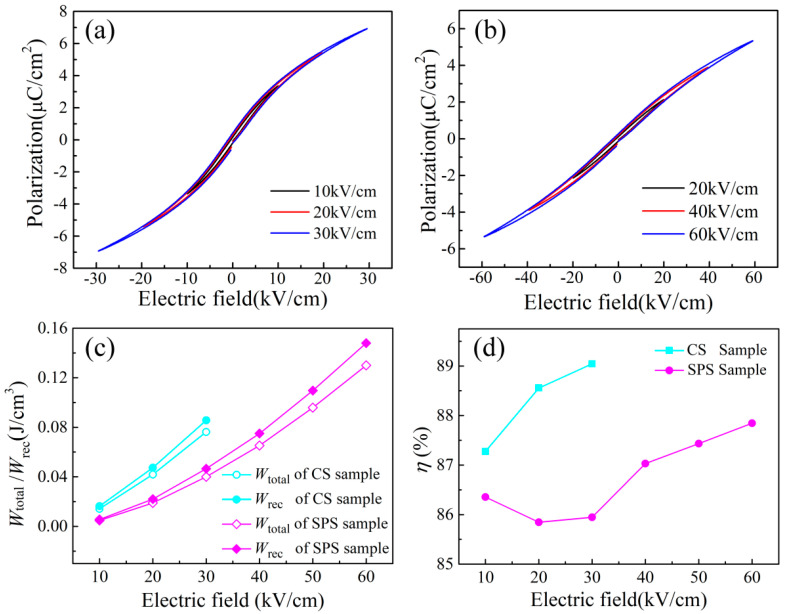
P-E loops measures with different electric fields of SBN60−2La prepared by (**a**) CS and (**b**) SPS; (**c**) *W*_total_, *W*_rec_ and (**d**) *η* of the CS sample and SPS sample.

As plotted in Figure 3a,b, the breakdown electric field of the SPS sample is much higher than the CS sample. Because the sample thickness in this work is thick (1 mm), the measurable electric field and the obtained breakdown strength is relatively low. For the CS sample, the polarization shows a rapid increase under a low electric field, limiting the enhancement of the energy storage performance to a great extent. Under the same electric field, the maximum polarization value (*P*_max_) of the SPS sample is a little smaller than that of CS sample. decreases less than that of The P-E loop of SPS sample is more slant than that of the CS sample, which indicates the delayed polarization saturation. When the applied electric field increases, the maximum polarization can achieve a higher value, which can enhance the energy storage properties [26]. The calculated *W*_total_, *W*_rec_ and *η* of the CS sample and SPS sample are shown in Figure 4c,d. It can be seen that the *W*_total_ and *W*_rec_ of the SPS sample are much higher than that of the CS sample, which can be attributed to the higher breakdown strength of the SPS sample. It is well known that the breakdown strength *E*_b_ is closely related with the grain size (Ga): *E*_b_ ∝ (Ga)^−a^, where the exponent value is 0.2~0.4 [27]. The *E*_b_ is inversely proportional to the grain size. Dense microstructure and small grain size are beneficial to the enhancement of *E*_b_. Hence, the decreased grain size and the denser microstructure of SBN60-2La prepared by SPS in this work make an important contribution to the improvement of *E*_b_. The energy storage efficiency, *η*, of the CS sample is a little higher than that of the SPS sample, which is the result of the fast polarization saturation. However, both the SBN60-2La ceramics prepared by CS and SPS obtain a high efficiency *η* value, which is higher than 85%. This can be attributed to the slim P-E loop caused by the relaxor feature of the La modifier. The electric parameters of the SBN60-2La ceramics prepared by CS and SPS are summarized in Table 1.

## 4. Conclusions

La-doped Sr_0.6_Ba_0.4_Nb_2_O_6_ ferroelectric ceramics were prepared by the conventional solid state reaction method (CS) and spark plasma sintering (SPS), respectively. The grain shape changes from a columnar to an isometric crystal and the grain size decreases obviously after spark plasma sintering. Both the CS sample and SPS sample show obvious frequency dispersion and a diffuseness value *γ* close to 2, which indicates their classical relaxor behavior. All the samples show slim P-E loops, which verify the relaxor behavior. Moreover, the SPS sample has a higher *E*_b_ and *W*_rec_ due to the smaller grain size and denser microstructure. All the samples show a high *η* value of over 85%. This work provides guidance for improving the energy storage properties through refining grain size by SPS for strontium barium niobate ferroelectric ceramics.

## Figures and Tables

**Table 1:** The electric parameters of SBN60-2La prepared by CS and SPS.

Ceramics	Relative Density	Mean Grain Size (μm)	*T*_m_/1 kHz (°C)	*γ*/1 kHz	*W*_total_ (J·cm^−3^)	*W*_rec_ (J·cm^−3^)	*η*
CS Sample	95.3%	6.12	−3	1.72	0.086	0.076	89.0%
SPS Sample	98.5%	1.22	20	1.63	0.130	0.148	87.8%

## Data Availability

Not applicable.
